# Synthesis of Neem-Oil-Infused Niosome and Starch Nanoparticle Coatings for Preserving the Quality of Strawberry Fruit

**DOI:** 10.3390/foods14111860

**Published:** 2025-05-23

**Authors:** Rahul Islam Barbhuiya, Charles Wroblewski, Sivaranjani Palanisamy Ravikumar, Jayasankar Subramanian, Abdallah Elsayed, Ashutosh Singh

**Affiliations:** 1 School of Engineering, University of Guelph, Guelph, ON N1G 2W1, Canada; rbarbhui@uoguelph.ca (R.I.B.); wroblewc@uoguelph.ca (C.W.); palaniss@uoguelph.ca (S.P.R.); aelsay01@uoguelph.ca (A.E.); 2 Department of Plant Agriculture, University of Guelph, Guelph, ON N1G 2W1, Canada; jsubrama@uoguelph.ca

**Keywords:** nanoparticles, niosomes, pea starch, preservation, post-harvest, strawberry, shelf-life

## Abstract

Strawberries face marketing challenges due to their short post-harvest shelf-life, largely impacted by shrivelling, weight loss, fungal decay, and mechanical damage. Neem oil (NO) is known for its shelf-life extension benefits; however, encapsulation is needed to maintain its efficacy. This study aimed to stabilize and encapsulate NO in a polymeric and lipid material to preserve the quality of strawberries stored at 4 ± 1 °C, 80 ± 2% RH for seven days. After seven days, the nanoparticle-coated fruits showed a weight loss of around 5.9% with niosomes and 8.9% with starch nanoparticles, while the control had a significant 32.45% weight loss. Additionally, both nanoparticle coatings significantly (*p* < 0.05) preserved fruit colour compared to the untreated control. The findings suggest that nanoparticle coatings could serve as an active agent in preserving the quality of strawberries within the food supply chain. The study provides valuable insights into post-harvest management and fruit preservation, showcasing the effectiveness of these coatings as active packaging solutions.

## 1. Introduction

Strawberries are non-climacteric fruit cultivated as highly valued fresh produce globally. However, they are prone to spoilage due to shrivelling, mechanical damage, and fungal decay influenced by high moisture content, soft texture, and microbial contamination [1]. Two of the most prevalent post-harvest diseases in strawberries are soft rot, caused by different *Rhizopus* species [2], and grey mould, caused by *B. cinerea* [3]. Furthermore, strawberries also experience weight loss, which reduces their market price and affects overall fruit quality. In general, these problems are managed by synthetic-chemical-based products, which are often considered not environmentally friendly and hazardous [4,5]. As a result, it is important to explore environmentally friendly alternative methods for maintaining the post-harvest quality of strawberries. Therefore, there is a growing interest in using safe, eco-friendly, and natural alternatives like plant-derived oils (PDOs).

Oils such as PDOs possess a wide range of biological activities, including nutritional, medicinal, and antimicrobial properties [6,7]. Neem oil (*Azadirachta indica*) is a PDO with several bioactive compounds, including limonoid, nimbanal, nimbione, gedunin, salannin, azadirachtin, and nimbolide [7]. These compounds possess strong bactericidal, insecticidal, antifungal, and antioxidant properties, making them a potential option for extending the shelf-life and preserving the quality of the strawberries. Neem oil (NO) has also been used for many decades in traditional medicine practices in the southern regions of Asia and Africa to treat human diseases such as inflammation, diabetes, and cancer with recommended dosages [8]. However, due to its photosensitive properties, high volatility, and tendency to degrade rapidly, NO requires proper encapsulation to enhance its effectiveness as a natural antimicrobial agent in the food industry. Additionally, in this study, NO was selected because of its sustainability, availability, and natural source.

In recent years, various encapsulation techniques have been developed to overcome the challenges associated with PDOs [9,10]. Nanoencapsulation is one of the techniques which offers several advantages, particularly in protecting the physicochemical properties of the PDOs. In nanoencapsulation, PDOs are enclosed within a nanoscale delivery system known as nanocapsules or nanocarriers, typically ranging from 1 to 100 nanometres in size [9,10]. The small size of the nanocarrier allows for a uniform distribution and a controlled release of the PDOs on the fruit’s surface [11]. Secondly, as particle size is reduced to the nanoscale, the surface-to-volume ratio increases, enabling greater contact between the nanoparticle and the fruit surface, resulting in a targeted delivery of PDOs to areas with microorganisms [12]. Furthermore, the structure of nanocarriers aids in preserving the PDO and reducing its rate of evaporation [13]. Therefore, in this study, to preserve the quality of strawberries and reduce post-harvest losses, the potential application of two environmentally friendly and biodegradable coatings composed of organic nanomaterials was evaluated.

In general, organic nanomaterials are classified into two broad categories: polymer-based and lipid-based [14]. They show several advantages over other conventional nanocarrier systems due to their well-defined structure, stability, biodegradability, biocompatibility, and capability for controlled release of active ingredients [15]. Therefore, this study investigates the potential application of an NO-infused lipid-based nanomaterial (niosomes) and an NO-infused polymeric nanomaterial (starch), developed in our previous study [7,16], in preserving the quality of strawberries.

Niosomes are a type of lipid-based organic nanomaterial composed of non-ionic surfactants. They are non-toxic and have a unique bilayer structure enabling them to encapsulate both hydrophilic and hydrophobic bioactive compounds, including NO [7]. Similarly, starch-based polymeric nanoparticles are another class of organic nanomaterials that exhibit considerable potential for encapsulating NO, indicating promising applications in agriculture [16,17]. Additionally, both the nanomaterials were synthesized using two distinct encapsulation techniques (see Section 2.1), aiming to protect the physicochemical properties of NO. The research also aims to showcase the potential of eco-friendly active packaging coatings in preserving strawberries and reducing waste. The findings are expected to offer valuable insights into post-harvest management and food preservation, assessing the effectiveness of these novel coating methods as active packaging solutions.

## 2. Materials and Methods

Strawberries of the brand Driscoll’s were purchased at a fully ripe stage from a local supermarket in Guelph, Ontario, Canada. Fruits with any physical damage or diseases were excluded, while the remaining fruits were standardized for similar size, shape, and uniform colour. The surface of the fruits was disinfected with 10-times-diluted hydrogen peroxide solution (30 mg/L), followed by rinsing with distilled water [17,18]. Pea starch (*Pisum sativum* L.) and neem oil (NO) were purchased from a local grocery store in Guelph, Canada. Polysorbate 80 (Tween 80), a dialysis membrane tube (12–14 kDa), PBS (phosphate-buffered saline), and soy lecithin (SL) were procured from Fisher Scientific (Fisher Scientific, Guelph, ON, Canada) and used without any treatment. Absolute ethanol (≥ 99.8%) was obtained from Fisher Scientific (Fisher Scientific, Canada). All reagents and solvents used were of analytical grade and did not require further purification. De-ionized (DI) double-distilled (DD) water was used for all experiments.

### 2.1. Preparation of the Neem-Oil-Infused Starch Nanoparticles and Niosomes

The NO-infused starch nanoparticles (NOISNPs) were synthesized using a rapid spray solvent–antisolvent nanoprecipitation technique, as described by Barbhuiya, Wroblewski, Ravikumar, Kaur, Routray, Subramanian, Elsayed, and Singh [16]. Briefly, native pea starch was mixed with DI water at a concentration of 21 mg/mL and heated in a boiling water bath with an upright condenser for 20 min. The mixture was continuously stirred using a Isotemp^®^ magnetic stirrer (Fisher Scientific, Guelph, Canada) to achieve proper gelatinization. The NO was added to the mixture at a concentration of 2.5 mg/mL, identified as the minimum effective concentration against common plant pathogens, as described by Barbhuiya, Wroblewski, Elsayed, Subramanian, Kaur, Routray, and Singh [7]. The gelatinized starch–oil mixture was then homogenized for 1 min and 30 sec at 14,900 rpm using a Polytron^®^ homogenizer (PT 1300 D). In the rapid spray process, water was used as the solvent and pure ethanol as the antisolvent. The homogenized oil–starch mixture was sprayed into ethanol (1:10) using a syringe pump (Pump 11 Elite, Harvard Apparatus) through an airbrush (1/8 NPS (M), Mastercraft) at a rate of 595 µL/min with a constant airflow of 5 L/min. The NOISNPs were then separated from the ethanol–water mixture by centrifuging at 3500 rpm for 10 min, followed by removal of the supernatant. The NOISNPs were redispersed in DI water and stored at −80 °C for 24 h. After freezing, they were dried over three days using a freeze dryer (Labconco, Kansas City, MO, USA) at −50 °C.

Similarly, NO-loaded niosomes were prepared via a thin-layer hydration method, with slight modifications, as described by Barbhuiya, Wroblewski, Elsayed, Subramanian, Kaur, Routray, and Singh [7]. Tween 80 (T80), a non-ionic solvent, and soy lecithin, a lipid, were selected as the wall material to encapsulate the NO. A 10 mL organic solvent mixture (chloroform and ethanol, 1:1) was used to dissolve the NO and the wall materials, ensuring thorough mixing. The solvents were then evaporated using an IKA RV 8 V-C rotary evaporator (Cole-Parmer^®^, Quebec City, QC, Canada) at 75 °C for 30 min. Following evaporation, PBS buffer (pH 7.4) was added at 60 °C (50 rpm, 30 min) to rehydrate the dried thin films. The samples were sonicated to achieve an even distribution of NO-loaded niosomes. The samples were stored at 4 °C for future experiments.

### 2.2. Post-Harvest Treatment

**Figure 1:**
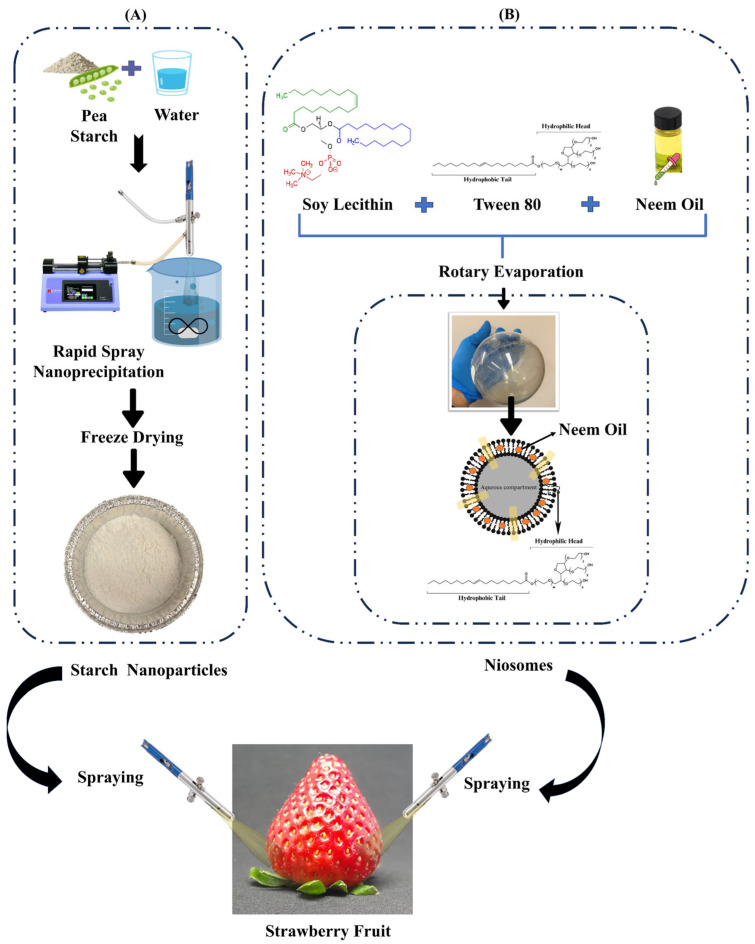
Schematic illustration of the post-harvest treatment of strawberries: (**A**) Top left box: Native pea starch (a polymer) was mixed with water to achieve proper gelatinization, followed by the addition of NO. The mixture was then processed using a rapid spray solvent–antisolvent nanoprecipitation method to synthesize NOISNPs. These nanoparticles were subsequently freeze-dried for storage and later re-dispersed in water before spraying onto the strawberries. (**B**) Top right box: NO-loaded niosomes were prepared using a thin-film hydration technique. A mixture of Tween 80 (a non-ionic surfactant), soy lecithin (a lipid), and NO was mixed in chloroform and ethanol to ensure proper mixing. A thin film was formed by rotary solvent evaporation and then rehydrated to produce niosomes. Both types of nanoparticles were finally applied to the surface of the strawberries through spraying.

Strawberries were randomly divided into three treatment groups, each containing three strawberries: (1) control (treated with water), (2) NOISNP-treated, and (3) niosome-treated. In brief, freeze-dried NOISNPs were re-dispersed in deionized (DI) water at a concentration of 21 mg/mL and then sprayed (as shown in Figure 1) onto the fruit surface from approximately ~10 cm at a rate of 2 mL/min, with an airflow of 5 L/min. This concentration was selected as it showed the highest encapsulation efficiency of ~82.6% [16]. Similarly, niosomes were sprayed at the same rate of 2 mL/min with an airflow of 5 L/min. The niosomes showed a maximum encapsulation efficiency of ~86.74% [7]. A single thin layer of coating was applied to each fruit. The fruits were then air-dried for 1 h.

Following air-drying, the strawberries were stored at 4 ± 1 °C for seven days in a 97990E model low-temperature refrigerated incubator (Fisher Scientific, Guelph, Canada), with treated and untreated samples placed on separate racks to prevent cross-contamination. The treatment groups were labelled as S1 (control), S2 (niosome-coated), and S3 (NOISNP-coated). Each treatment and the control were performed in triplicate. Temperature and humidity were recorded during sampling days using an RHM15 temperature and relative humidity meter (Extech Instruments, Guelph, ON, Canada). The storage conditions (4 ± 1 °C and 80 ± 2% RH) were selected to closely mimic typical retail and consumer refrigeration environments [19]. Furthermore, the study was discontinued after seven days because the control sample had shrivelled and was no longer suitable for further testing. The physicochemical properties of the fruits, such as colour, total soluble solids, pH, antioxidant activity, total phenolic content, and visual inspection, were monitored and compared over the seven-day storage period.

### 2.3. Weight Loss, Total Soluble Solids (TSS), and pH

Fruits from each treatment were weighed on a digital balance initially and after each storage period (three and seven days). Total weight loss was determined by calculating the difference between initial and final weights, expressed as a percentage [20]. Total soluble solids (TSS) in the fruit extract were measured with a MA871 digital refractometer (Milwaukee, Milwaukee, WI, USA) by placing a drop (~0.05 mL) of juice from each fruit, representing three replicates per treatment, onto the prism glass for a %TSS reading, expressed as %Brix. The refractometer was calibrated with DI water for a 0% reading before each analysis. To assess pH, fruits from each replicate and treatment were homogenized at 3000 rpm for 2 min with a handheld homogenizer. The juice was then centrifuged at 3000 rpm to remove coarse fibres, and the supernatant was filtered through Whatman filter paper. The pH was measured using a Star A326 digital pH meter from Thermo-Scientific (Guelph, ON, Canada).

### 2.4. Total Phenolic Content

The total phenolic content (TPC) of the strawberry extract was determined using a modified Folin–Ciocalteu method [21,22]. In brief, 1 g of strawberry pulp was mixed with 10 mL of methanol and incubated at 50 °C for 60 min. A 2 mL sample of the mixture was centrifuged at 3000 rpm for 5 min, and the supernatant was collected. Then, 1 mL of the supernatant was mixed with 7.5 mL of DI water and 0.5 mL of Folin–Ciocalteu reagent. The mixture was left at room temperature (22 ± 2 °C) for 5 min before adding 1 mL of 5% (*w*/*v*) sodium bicarbonate. After a 90 min incubation, 200 µL of the solution was transferred to a 96-well plate, and absorbance was measured at 765 nm. TPC was reported as milligrams of gallic acid equivalents (GAE) per gram of fresh weight (FW).

### 2.5. Antioxidant Activity

The antioxidant activity of the fruits was evaluated using the 2,2-diphenyl-1-picrylhydrazyl (DPPH) radical scavenging assay with some modifications [16,23]. In brief, 1.5 mL of a 1 mM methanolic DPPH solution was combined with 50 µL of the methanolic extract and incubated for 20 min in the dark at room temperature (22 ± 2 °C). The antioxidants in the fruit extract reacted with the free radicals in the DPPH, leading to a reduction in the purple colour. The resulting decrease in absorbance, indicating the fading of the purple colour, was measured at 517 nm using a Genesys 10S UV-spectrophotometer from Thermo-Scientific (Guelph, Canada). The antioxidant activity was then calculated using Equation (1) [23]:

(1) $$Antioxidant\;activity\left(\%\right)=\frac{Absorbance\;of\;blank-Absorbance\;of\;sample}{Absorbance\;of\;blank}\times 100$$

### 2.6. Colour Measurement

A handheld colorimeter (CR20, Konica Minolta, Ramsey, NJ, USA) was used to measure the colour change of the strawberries [24]. The colour analysis was performed at three locations: the top, centre, and bottom. Colour assessment was performed using the CIELab* and CIELCh° colour scales, where L* represents brightness (0 = black; 100 = white), a* represents green (−) to red (+), and b* from blue (−) to yellow (+). Chroma (C*) and hue angle (h°) were calculated based on a* and b* values using Equations (2) and (3) [25].

(2) $$C^{*}={\left(a^{*2}+b^{*2}\right)}^{0.5}$$

(3) $$h^{{}^{\circ}}=\arctan\left(b^{*}/a^{*}\right)$$

### 2.7. Statistical Analysis

To ensure reproducibility, each experiment was conducted in triplicate, with the results presented as means ± standard deviation. Variance analysis was carried out using IBM SPSS Statistics 27 at a 5% significance level. Student’s *t*-test was applied for comparisons between two groups, while analysis of variance (ANOVA) was used for comparisons among multiple groups. A *p*-value of less than 0.05 was considered statistically significant.

## 3. Results and Discussion

### 3.1. Weight Loss

Weight loss in fruits is primarily linked to respiration and the evaporation of moisture through the skin. Strawberries are susceptible to weight loss due to their thin protective skin [20]. Therefore, one of the key contributors to the perishability of strawberries is the rapid water loss from their surface [20], which results in wrinkling, spoilage, and dehydration, followed by shrinkage and quality deterioration. In this study, nearly all fruits showed weight loss (Table 1), with significant differences (*p* < 0.05) observed in weight loss percentages among the treatments and control by day seven of storage. Control samples experienced the highest weight loss, reaching 21.4 ± 2.8% by day three and 32.45 ± 2.4% by day seven. In contrast, fruits coated with niosomes showed the lowest weight loss, at 2.17 ± 1.12% after three days and 5.9 ± 1.50% by day seven, followed by NOISNP-coated fruits with a weight loss of 3.88 ± 3.2% after three days and 8.9 ± 3.02% by day seven. Weight loss in fruits generally occurs through the skin, driven by a vapour pressure gradient between the fruit and the environment, which accelerates ripening, softening, and senescence due to metabolic processes like ethylene production [26]. In this study, the reduced weight loss in coated fruits can be attributed to the semipermeable barrier formed by the polymer (starch) or lipid (niosomes) coating, which limits the exchange of moisture, carbon dioxide, and oxygen, thereby slowing water loss, respiration, and oxidation [27,28]. These results are consistent with findings by Ansarifar and Moradinezhad [1], where strawberries treated with Zein fibre film infused with thyme essential oil had significantly reduced weight loss compared to the control. Similarly, da Silva Bruni et al. [29] reported that a κ-carrageenan/starch-based coating produced a barrier effect on the fruit surface, which delayed water loss and transpiration rates.

### 3.2. Total Soluble Solids and pH

Total soluble solids and pH are the two key indicators used to assess the maturity and flavour of strawberries [30]. It is the sugar content and organic acids, which contributes to the fruit’s sweet taste [29,31]. Similarly, the pH plays an essential role in determining the ripening and taste of strawberries [32].

In this study, the total soluble solids content in strawberries increased throughout the ripening process for all fruit samples, indicating the increased metabolic activity of ripe strawberries [3]. Specifically, untreated strawberries showed the highest total soluble solid levels during ripening (Table 1). The total soluble solid values rose from an initial 7.2 ± 0.1 °Brix to a final 12.5 ± 0.2 °Brix in the control group. However, by the end of the seven days, the niosome-treated samples had lower total soluble solid levels (9.2 ± 0.1 °Brix) compared to the NOISNPs (10.1 ± 0.1 °Brix) and the control, likely due to the coating slowing the ripening process, which may have led to fruit expansion, hindering carbohydrate transport and resulting in reduced sugar content [33]. Bahmani et al. [34] suggested that the rise in total soluble solids could be linked to the breakdown of starch into soluble sugars like glucose and sucrose. This process is part of the fruit’s physiological or metabolic activity and contributes to enhanced flavour and sweetness.

Similarly, the pH of control strawberries (Table 1) showed an increasing trend from 3.3 ± 0.02 to 3.6 ± 0.01, while the treated samples remained relatively stable at 3.4 ± 0.03. The increase in the control may be attributed to continuous water loss and the generation of organic acids due to respiratory by-products, raising acidity over time [35]. The results of weight loss and total soluble solids analysis also confirmed this observation. After seven days, the control strawberries showed a higher pH, potentially due to alkaline autolysis compounds (e.g., nitrogen-containing substances) that form as strawberries deteriorate, contributing to the rise in pH [36]. Similar findings were reported by Gol et al. [37], who reported an increase in pH in control samples compared to coated strawberries. Jiang et al. [38] and Alharaty and Ramaswamy [39] have also shown that coatings made from chitosan, sodium alginate, and calcium chloride helped slow down changes in pH levels in strawberries, thereby delaying ripening, inhibiting fungal growth, and preventing early fruit deterioration. Moreover, since only minor differences were observed in the pH and total soluble solids between the treated samples and the untreated controls, it can be inferred that the coating might not have impacted the sensory characteristics of the fruit.

### 3.3. Colour Change and Visual Assessment

**Figure 2:**
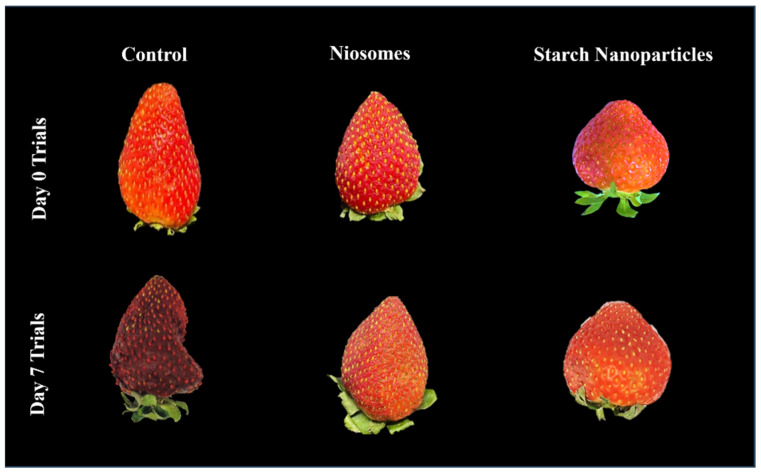
The colour change and appearance of strawberry fruits were influenced by various treatments after seven days of storage.

The red colour of strawberry fruit is an important quality factor that significantly impacts its marketability and ripeness [40]. Table 2 and Figure 2 provide an overview of colour parameter changes in strawberries treated with various coatings. For all treatments, the L* values decreased with the storage period. The a* and b* values increased in the treated samples, indicating retained colour; however, these values decreased in the control, which might be due to darkening of the fruit. Strawberries treated with both niosomes and NOISNPs showed only very small changes in colour parameters (L*, a*, b*, hue angle, and chroma) throughout storage, indicating the effectiveness of the coating in preserving colour and quality. The results of pH and total soluble solids analysis confirmed this observation. These results align with findings by Wani et al. [41] and Ansarifar and Moradinezhad [1], which showed that post-harvest treatments prolonged strawberry shelf-life and maintained a lighter colour than untreated samples. Additionally, no signs of fungal infection were observed in any samples, likely due to initial hydrogen peroxide washing and cold storage conditions. Overall, the control samples showed faster colour change and increased wrinkling, whereas the treated samples better preserved the colour and appearance.

### 3.4. Antioxidant Activity and Total Phenolic Content

Antioxidant activity and total phenolic content are two important quality indicators for strawberries that reflect their capacity to resist oxidative stress during storage [42]. This study showed that the antioxidant activity of strawberries decreased significantly (*p* < 0.05) with the storage period for the treated samples. However, the antioxidant activity of the control at first showed a decrease at day three (71 ± 0.1%) then an increase at day seven (79.33 ± 0.57%). The variation could have resulted from oxidative stress reducing antioxidants, followed by an increase later due to over-ripening leading to the production of additional phenolic compounds [43,44]. Furthermore, after seven days of storage, the scavenging activity in fruits treated with niosomes was higher (68.66 ± 0.58%) than that of fruits treated with NOISNPs (66.66 ± 0.55%), with both showing lower activity compared to the control (79.33 ± 0.57%). This suggests both treatments effectively scavenged DPPH free radicals and helped delay the ripening of the fruits. The results align with Wani, Gull, Ahad, Malik, Ganaie, Masoodi, and Gani [41], who reported a decrease in antioxidant activity in strawberries with edible coatings maintaining higher activity compared to untreated controls, and with Taheri et al. [45], who highlighted the strong antioxidant properties of essential oils in reducing oxidative stress.

**Figure 3:**
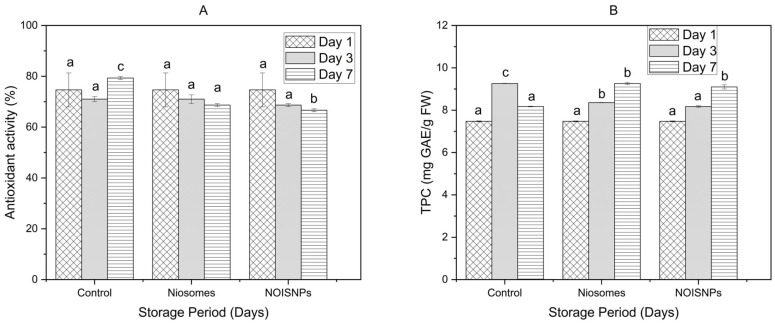
Graphical representation for (**A**) antioxidant activity and (**B**) total phenolic content of strawberry fruits over a seven-day storage period for both control and treated samples. The data points represent the mean values (*n* = 3) ± standard deviation. Different lowercase letters (e.g., a, b, c) indicate statistically significant differences between treatment means at *p* < 0.05. Means that share the same letter are not significantly different from each other.

Similarly, the total phenolic content increased in all treated fruit samples (Figure 3). On day three, the control fruits had the highest total phenolic content (9.26 ± 0.01 mg GAE/g FW), while fruits treated with NOISNPs had the lowest (8.17 ± 0.04 mg GAE/g FW). By day seven, niosome-treated fruits showed the highest total phenolic content (9.26 ± 0.04 mg GAE/g FW), while control fruits exhibited the lowest (8.17 ± 0.01 mg GAE/g FW). Lower phenolic content in control samples, compared to treated ones, likely results from a higher respiration rate that accelerates phenolic breakdown, suggesting that both types of nanoparticles may help preserve total phenolic content in strawberries during post-harvest storage [46]. Generally, phenolic content is affected by both the number and position of OH groups, which enhance antioxidant activity by donating hydrogen to free radicals, thereby improving scavenging effects [47]. However, DPPH radical scavenging is not solely determined by total phenolic content; other antioxidants, such as ascorbic acid, can also contribute [48]. The breakdown of antioxidant compounds into smaller molecules can reduce DPPH scavenging capacity and overall antioxidant activity. Thus, it was observed that while antioxidant activity in the samples decreased, total phenolic content increased.

## 4. Conclusions

This study demonstrated that encapsulating NO in niosomes and NOISNPs shows promise as an eco-friendly active packaging solution that can effectively preserve the quality and extend the post-harvest shelf-life of strawberries. The shelf-life analysis showed that both types of nanoparticles delayed ripening while maintaining desirable pH, total soluble solids, weight, and colour compared to the control. In untreated fruits, bioactive compounds degraded more quickly, causing tissue damage and cell membrane loss, leading to reduced colour and weight. However, strawberries treated with niosomes retained better quality characteristics than those treated with NOISNPs and the control group. In addition, coated fruits showed no signs of microbial spoilage, likely due to initial washing with hydrogen peroxide and cold storage. In conclusion, these coatings effectively sustain storage quality and extend the shelf-life of perishable fruits, presenting a safe and effective alternative to harmful chemical additives in food preservation. Both types of coatings are practical and environmentally friendly, being biodegradable, widely available, and easy to use. This combination offers a promising active packaging solution for prolonging the shelf-life of fruits and vegetables. Future research will focus on optimizing various concentrations of the nanomaterials, evaluating their performance under different storage conditions, and investigating different PDOs and nanocarrier systems to further improve the shelf-life and quality of various fruits. Additionally, the effectiveness of these nanomaterials will be assessed on plants grown in a greenhouse environment.

## Figures and Tables

**Table 1:** Quality parameters of strawberry fruit over a seven-day storage period: (a) weight loss percentage; (b) total soluble solid content (% Brix); (c) pH, for both control and treated samples. The data points represent the mean values (*n* = 3) ± standard deviation. Different lowercase letters (e.g., a, b, c) indicate statistically significant differences between treatment means at *p* < 0.05. Means that share the same letter are not significantly different from each other.

Treatments	Day 1	Day 3	Day 7
	Weight loss (%)		
Control	NA	21.4 ± 2.8 b	32.45 ± 2.4 b
Niosome-treated	NA	2.17 ± 1.12 a	5.9 ± 1.50 a
Starch-nanoparticle-treated	NA	3.88 ± 3.2 a	8.9 ± 3.02 a
	Total soluble solids (°Brix)		
Control	7.2 ± 0.1 a	10 ± 0.2 b	12.5 ± 0.2 c
Niosome-treated	7.2 ± 0.1 a	9.2 ± 0.1 a	9.2 ± 0.1 a
Starch-nanoparticle-treated	7.2 ± 0.1 a	9.7 ± 0.01 b	10.1 ± 0.1 b
	pH		
Control	3.3 ± 0.02 a	3.5 ± 0.01 b	3.6 ± 0.01 b
Niosome-treated	3.3 ± 0.02 a	3.4 ± 0.01 a	3.4 ± 0.01 a
Starch-nanoparticle-treated	3.3 ± 0.02 a	3.4 ± 0.02 a	3.4 ± 0.03 a

**Table 2:** Colour parameters of strawberries fruit over a seven-day storage period: (a) L* scale (0 = black; 100 = white), (b) a* scale [green (−) to red (+)], (c) b* scale [blue (−) to yellow (+)], (d) chroma (C*), and (e) hue angle (h°) for both control and treated samples. Different lowercase letters (e.g., a, b, c) indicate statistically significant differences between treatment means at *p* < 0.05. Means that share the same letter are not significantly different from each other.

Treatment	Days	L*	a*	b*	Chroma	Hue
Control	1	28.33 ± 1.70 b	29.86 ± 4.75 a	17.7 ± 3.64 a	34.76 ± 5.81 a	30.43 ± 1.46
Niosome-treated	1	32.47 ± 6.40 b	19.50 ± 8.85 a	19.26 ± 2.49 a	33.66 ± 8.5 a	28.47 ± 1.82
Starch-nanoparticle-treated	1	29.63 ± 7.35 a	22.77 ± 1.77 a	15.80 ± 1.73 a	29.53 ± 5.87 a	32.23 ± 2.35
Control	3	21.10 ± 1.28 a	20.77 ± 0.91 a	9.53 ± 1.46 a	22.93 ± 0.57 a	24.60 ± 4.13 a
Niosome-treated	3	27.40 ± 4.30 a	27.73 ± 6.27 a	14.03 ± 3.25 a	29.36 ± 6.4 a	28.46 ± 1.82 a
Starch-nanoparticle-treated	3	26.87 ± 2.32 a	24.37 ± 1.40 a	13.37 ± 2.08 a	27.80 ± 2.23 a	28.67 ± 2.39 a
Control	7	20.90 ± 3.53 a	16.30 ± 0.53 a	7.57 ± 1.00 a	18.00 ± 0.61 a	24.80 ± 3.12 a
Niosome-treated	7	26.60 ± 4.76 c	29.90 ± 4.29 b	27.50 ± 1.15 c	44.27 ± 3.30 c	41.60 ± 1.15 c
Starch-nanoparticle-treated	7	19.10 ± 0.40 ab	25.63 ± 0.31 b	19.87 ± 0.21 b	31.73 ± 0.61 b	36.30 ± 1.51 b

## Data Availability

Data will be available upon request.
